# Low Maternal Vitamin B12 Status Is Associated with Lower Cord Blood HDL Cholesterol in White Caucasians Living in the UK

**DOI:** 10.3390/nu7042401

**Published:** 2015-04-02

**Authors:** Antonysunil Adaikalakoteswari, Manu Vatish, Alexander Lawson, Catherine Wood, Kavitha Sivakumar, Philip G. McTernan, Craig Webster, Neil Anderson, Chittaranjan S. Yajnik, Gyanendra Tripathi, Ponnusamy Saravanan

**Affiliations:** 1 Warwick Medical School, University of Warwick, Coventry CV2 2DX, UK; E-Mails: A.K.Antonysunil@warwick.ac.uk (A.A.); K.Sivakumar@warwick.ac.uk (K.S.); P.G.McTernan@warwick.ac.uk (P.G.M.); 2 Nuffield Department of Obstetrics & Gynaecology, University of Oxford, Oxford OX3 9DU, UK; E-Mail: manu.vatish@obs-gyn.ox.ac.uk; 3 Department of Pathology, Heartlands Hospital, Birmingham B9 5SS, UK; E-Mails: alexander.lawson@heartofengland.nhs.uk (A.L.); craig.webster@heartofengland.nhs.uk (C.W.); 4 Academic department of Diabetes and Metabolism, George Eliot Hospital, Nuneaton CV10 7DJ, UK; E-Mails: Catherine.Wood@geh.nhs.uk (C.W.); neil.anderson@geh.nhs.uk (N.A.); 5 Diabetes Research Centre, KEM Hospital, Pune 411011, India; E-Mail: csyajnik@hotmail.com; 6 WISDEM Centre, University Hospital Coventry and Warwickshire, Coventry CV2 2DX, UK

**Keywords:** vitamin B12, maternal, offspring, metabolic risk, lipids

## Abstract

Background and Aims: Studies in South Asian population show that low maternal vitamin B12 associates with insulin resistance and small for gestational age in the offspring. Low vitamin B12 status is attributed to vegetarianism in these populations. It is not known whether low B12 status is associated with metabolic risk of the offspring in whites, where the childhood metabolic disorders are increasing rapidly. Here, we studied whether maternal B12 levels associate with metabolic risk of the offspring at birth. Methods: This is a cross-sectional study of 91 mother-infant pairs (*n* = 182), of white Caucasian origin living in the UK. Blood samples were collected from white pregnant women at delivery and their newborns (cord blood). Serum vitamin B12, folate, homocysteine as well as the relevant metabolic risk factors were measured. Results: The prevalence of low serum vitamin B12 (<191 ng/L) and folate (<4.6 μg/L) were 40% and 11%, respectively. Maternal B12 was inversely associated with offspring’s Homeostasis Model Assessment 2-Insulin Resistance (HOMA-IR), triglycerides, homocysteine and positively with HDL-cholesterol after adjusting for age and BMI. In regression analysis, after adjusting for likely confounders, maternal B12 is independently associated with neonatal HDL-cholesterol and homocysteine but not triglycerides or HOMA-IR. Conclusions: Our study shows that low B12 status is common in white women and is independently associated with adverse cord blood cholesterol.

## 1. Introduction

The prevalence of childhood obesity is increasing rapidly [1,2]. Recently, the Early Childhood Longitudinal Study demonstrated that 27.3% of children were either overweight or obese by the time they enter kindergarten in the United States [1]. Higher rate of childhood obesity is a likely contributor for the increasing incidence of type 2 diabetes (T2D) earlier in life as well as pre-gestational and gestational diabetes (GDM) in women [3]. It is known that childhood obesity independently predicts obesity and metabolic disorders in the adulthood [4]. Children born with lower HDL and higher triglyceride levels were small for gestational age (SGA) and had higher abdominal circumference [5]. It is known that both higher abdominal circumference and SGA are associated with future development of T2D and GDM [6,7] in many populations.

Although current adverse lifestyle (nutrition and physical inactivity) contributes to obesity, a growing body of evidence links nutrient imbalance in early life to the development of metabolic disorders in childhood and in adults [8]. Many studies support this link including the Dutch-famine study. Individuals exposed to nutritional imbalance during pregnancy are likely to be obese, have early onset of coronary artery disease, T2D and worse cognitive performances as adults [9]. Emerging evidence from clinical studies show that key maternal micronutrients involved in the one-carbon metabolism (1-C) can cause adverse metabolic programming. Independent studies from South Asia have demonstrated that children born to mothers with low vitamin B12 [10,11] and higher folate [12] have greater insulin resistance. In addition, low maternal B12 levels independently contributed to the risk of small for gestational age (SGA), which has been shown to increase the metabolic risk of the offspring [13]. Vegetarianism is the likely cause of high prevalence of low B12 levels in these population [14]. In a Brazilian pregnancy cohort, low maternal B12 was associated with lower levels of the methyl donor (*S*-adenosyl methionine—SAM) in the cord blood [15]. A study in a Chinese population demonstrated that low maternal B12 is common during pregnancy and is associated with an altered methylation pattern of the insulin growth factor 2 (IGF2) promoter region in the cord blood [16], highlighting a potential role of B12 on fetal growth. Further, animal studies showed that maternal vitamin B12 deficiency resulted in higher adiposity, insulin resistance, blood pressure [17] and adverse lipid profile in the offspring [18,19]. These investigations provide evidence that low maternal B12 could be an independent determinant of adverse metabolic phenotypes in the offspring.

Recently, we demonstrated in Europeans and Indians with T2D that vitamin B12 deficiency is associated with adverse lipid profile [20]. Re-analysis of the UK National Diet and Nutrition Survey data showed that low vitamin B12 levels (<191 ng/L) is common in the adult population (10%) and in women of reproductive age (14%) [21]. Our preliminary study of white pregnant women showed that the rate of low B12 status was as high as 20% at 16–18 weeks of gestation [22].

Despite the evidence that vitamin B12 deficiency is a potential contributor for adverse offspring metabolic phenotypes and the prevalence of low B12 status is increasing in White Caucasian population, the link between maternal B12 status and metabolic risk at birth is unexplored in the White Caucasian population. Therefore, the objective of our study was to investigate whether maternal B12 levels in white women independently associate with the metabolic risk at birth.

## 2. Methods

### 2.1. Study Population

The study was conducted in University Hospital Coventry Warwickshire (UHCW), Coventry, UK. All study participants were pregnant women delivering at 39–40 weeks of gestation. The Coventry local research ethics committee approved the study, and all patients gave written informed consent (Research Ethics Committees 07/H1210/141). Women with known chronic diseases were excluded. Maternal data including parity, smoking, BMI and birth weight were collected from pregnancy records. Folic acid supplement use collected but detailed dietary history was not recorded. Maternal BMI measured routinely at the first pregnancy visit (before 10 weeks of gestation). We collected 182 maternal venous and cord blood samples (91 mother-newborn pairs) at the time of delivery. Extrapolating from our preliminary studies [21,22], we anticipated around 20%–25% of the mothers to have low levels of vitamin B12 (<191 ng/L). To detect a similar proportion of low B12 status a sample size of 100–120 was required. The samples were collected in the fasting state, in tubes without anticoagulant and centrifuged at 2000 rpm/10 min. Serum was separated, aliquoted and stored at −80 °C until analysis.

### 2.2. Analytical Determinations

Serum glucose, cholesterol, triglycerides, HDL cholesterol were determined using an auto analyser Synchron CX7 (Beckman Coulter, Fullerton, CA, USA) based on enzymatic colorimetric assays. Insulin was measured using Invitrogen ELISA kit (Camarillo, CA, USA) according to manufacturer’s instructions. LDL cholesterol was calculated using Friedewald formula. Insulin resistance (HOMA-IR) was calculated by the Homeostasis Model Assessment 2 computer model (HOMA2) using fasting insulin and glucose levels [10]. Serum B12 and folate were determined by electrochemiluminescent immunoassay using a Roche Cobas immunoassay analyzer (Roche Diagnostics UK, Burgess Hill, UK). Similar to other studies [20,23,24], we have used 191–663 ng/L for serum Vitamin B12 and 4.6–18.7 μg/L for serum folate as normal range, respectively. The inter-assay coefficient of variations for B12 and folate were 3.9% and 3.7%, respectively. To avoid potential bias, all the biochemical analyses were conducted in a single batch to minimise assay variation. All the laboratory personnel were blinded and did not have any access to the clinical data. Serum homocysteine was determined by stable isotopic dilution analysis liquid chromatography (LC-MS/MS) [25] using a Waters Equity UPLC system (Waters, Milford, CT, USA) coupled to an API 4000 tandem mass spectrometer (Applied Biosystems, Warrington, UK). Due to the uncertainty of defining deficiencies of serum vitamin B12 and folate levels during pregnancy and cord blood, the terms “low B12 status” and “low folate status” were used throughout the manuscript if the levels were below 191 ng/L and 4.6 μg/L, respectively.

### 2.3. Statistical Analysis

Continuous data are reported either as mean ± standard deviation (SD) or geometric mean with 95% confidence intervals (CI). Categorical data are reported in numbers (percentages). The distributions of the maternal and neonatal parameters such as vitamin B12, folate, cholesterol, triglycerides, HDL, LDL, glucose, insulin, HOMA-IR and homocysteine concentrations were skewed; these data were log-transformed before analyses. Student’s *t*-test was used for comparison of groups. Bivariate correlations were done using Pearson test. Variables that showed significant associations with dependent variable (neonatal metabolic risk factors) were included as independent variables in the multiple linear regression analyses. To facilitate comparison, dependent and independent variables were converted into standard deviation scores (SDS). The data are presented as SD change in offspring outcome per SD change in maternal vitamin B12, folate and homocysteine. Associations between maternal vitamin B12, folate and homocysteine concentrations and offspring outcomes were examined in multivariate linear regression using 3 models. Model 1: unadjusted; Model 2: adjusted for maternal age, BMI, glucose, insulin, parity, folic acid supplement use, smoking, vitamin B12, folate and homocysteine; Model 3: Model 2 + respective maternal variable. All tests were two-sided, and *p* values of <0.05 were considered to be statistically significant. All analyses were performed using SPSS Statistics version 21 (IBM Corp, Armonk, NY, USA).

## 3. Results

### 3.1. B12, Folate and Homocysteine Status

The clinical characteristics of mothers and neonates are shown in Table 1. The prevalence of serum low vitamin B12 and folate status in women during pregnancy were 40% and 11% in mothers and 29% and 0% in neonates, respectively (Table 1). In cord blood, all the biochemical parameters were significantly lower than in maternal serum, except for the B12 and folate levels (Table 1). Children born to mothers with low B12 status had significantly lower B12 levels compared to those born to mothers with normal levels (Table 2). Mothers with higher parity and smoking had lower B12 levels. Those with self-reported folic acid supplement use had higher B12 and lower homocysteine levels (Table 3). Maternal B12, folate and homocysteine showed strong positive correlation with the respective offspring indices (B12: *r* = 0.648, folate: *r* = 0.706, homocysteine: *r* = 0.756, all *p* < 0.0001) (Supplementary Figure S1a–c). Neonatal homocysteine showed negative correlation with maternal B12 and folate (B12: *r* = −0.409, *p* < 0.0001; folate: *r* = −0.346, *p* < 0.001; Supplementary Figure S2a,b).

**Table 1:** Clinical characteristics of mothers and neonate.

	Mother	Neonate
	*n* = 91	*n* = 91
Age (years)	32.7 ± 5.9 ^a^	-
Weight (Kg)	77.7 ± 18.1	3.57 ± 0.26
Height (m)	1.62 ± 0.09	-
BMI (early pregnancy) (kg/m^2^)	29.4 ± 6.2	-
Glucose (mmol/L)	4.37 ± 0.42	3.88 ± 0.52
Insulin (mIU/L)	11.6 (12.9, 17.4) ^b^	8.01 (8.62, 11.9)
Triglycerides (mmol/L)	2.69 (2.62, 3.06)	0.23 (0.22, 0.26)
Cholesterol (mmol/L)	6.48 (6.31, 6.89)	1.68 (1.63, 1.84)
LDL cholesterol (mmol/L)	3.53 (3.46, 3.95)	0.82 (0.79, 0.95)
HDL cholesterol (mmol/L)	1.56 (1.53, 1.72)	0.74 (0.72, 0.83)
HOMA-IR	1.37 (1.55, 2.09)	0.99 (1.04, 1.41)
Vitamin B12 (ng/L)	218 (213, 289)	290 (292, 418)
Low B12 status (%)	36 (40) ^c^	26 (29)
Folate (μg/L)	10.5 (10.9, 13.2)	16.8 (16.4, 17.7)
Low folate status (%)	10 (11)	0
Homocysteine (μmol/L)	6.23 (6.02, 7.54)	5.76 (5.64, 6.85)

^a^ Mean ± SD (all such values); ^b^ Geometric mean (95% CI) (all such values); ^c^ Numbers (percentages) (all such values).

**Table 2:** Clinical characteristics of mothers and neonate according to maternal B12 levels.

	Mothers		Neonate	
	Maternal B12 ≥191 (ng/L)	Maternal B12 <191 (ng/L)	Maternal B12 ≥191 (ng/L)	Maternal B12 <191 (ng/L)
	*n* = 55	*n* = 36	*n* = 55	*n* = 36
Age (years)	33.0 ± 6.2 ^a^	32.3 ± 5.6	-	-
Weight (Kg)	74.3 ± 15.8	82.9 ± 20.8 *	3.58 ± 0.31	3.57 ± 0.18
Height (m)	1.62 ± 0.07	1.61 ± 0.11	-	-
BMI (early pregnancy) (kg/m^2^)	28.4 ± 6.1	30.8 ± 6.4 *	-	-
Glucose (mmol/L)	4.40 ± 0.46	4.36 ± 0.34	3.85 ± 0.52	3.94 ± 0.52
Insulin (mIU/L)	10.4 (11.7, 18.0) ^b^	13.7 (12.7, 18.6)	8.27 (8.33, 12.75)	7.64 (7.30, 12.40)
Triglycerides (mmol/L)	2.49 (2.37, 2.93)	3.04 (2.82, 3.48) *	0.21 (0.20, 0.24)	0.26 (0.23, 0.32) **
Cholesterol (mmol/L)	6.23 (5.99, 6.72)	6.86 (6.51, 7.43) *	1.72 (1.64, 1.92)	1.62 (1.51, 1.83)
LDL cholesterol (mmol/L)	3.29 (3.16, 3.82)	3.91 (3.67, 4.38) *	0.79 (0.76, 0.90)	0.87 (0.77, 1.09)
HDL cholesterol (mmol/L)	1.61 (1.54, 1.80)	1.49 (1.42, 1.70)	0.79 (0.76, 0.91)	0.67 (0.62, 0.77) *
HOMA-IR	1.18 (1.37, 2.14)	1.68 (1.57, 2.27)	0.94 (0.95, 1.41)	1.08 (0.98, 1.63)
Vitamin B12 (ng/L)	288 (265, 378)	146 (139, 155) ***	367 (354, 544)	202 (187, 234) ***
Low B12 status (%)	0	36 (40) ^c^	8 (14.5)	18 (50)
Folate (μg/L)	11.7 (11.5, 14.6)	9.0 (8.5, 12.4) *	17.6 (17.0, 18.5)	15.7 (14.9, 17.2) **
Low folate status (%)	3 (5.5)	7 (19.4)	0	0
Homocysteine (μmol/L)	5.50 (5.26, 6.18)	7.53 (6.69, 10.1) ***	4.97 (4.74, 5.59)	7.09 (6.58, 8.96) ***

^a^ Mean ± SD (all such values); ^b^ Geometric mean (95% CI) (all such values); ^c^ Numbers (percentages) (all such values); * *p*-value compared to maternal B12 (≥191 ng/L) group; * *p* < 0.05; ** *p* < 0.01; *** *p* < 0.001.

**Table 3:** Vitamin B12, folate and homocysteine in mothers and neonate according to maternal smoking status, parity and folate supplement use.

	Smoking	*n* = 91	Vitamin B12 (ng/L)	Folate (μg/L)	Homocysteine (μmol/L)
**Maternal**	No (%)	55	245 (227, 357)	10.7 (10.6, 13.9)	6.15 (5.61, 8.08)
	Yes (%)	45	189 (176, 224) **	10.1 (9.7, 13.3)	6.33 (5.90, 7.45)
**Neonate**	No (%)	55	327 (305, 502)	16.9 (16.4, 18.2)	5.42 (5.06, 6.58)
	Yes (%)	45	252 (232, 364) *	16.5 (15.8, 17.7)	6.21 (5.80, 7.68)
	**Parity**				
**Maternal**	Para 0 (%)	18	248 (203, 332)	13.7 (11.8, 17.9)	6.25 (5.21, 8.03)
	Para 1 (%)	48	224 (201, 347)	11.1 (10.8, 14.1)	6.12 (5.73, 7.43)
	Para ≥2 (%)	34	195 (179, 239) *	8.2 (7.6, 11.6) **	6.4 (5.31, 8.97)
**Neonate**	Para 0 (%)	18	327 (264, 479)	17.9 (16.6, 19.6)	5.61 (4.50, 7.59)
	Para 1 (%)	48	284 (258, 487)	16.9 (16.3, 18.1)	5.91 (5.53, 7.30)
	Para ≥2 (%)	34	283 (250, 396)	16.0 (15.2, 17.4) *	5.67 (5.08, 7.15)
	**Folate supplement users**				
**Maternal**	Yes (%)	85	224 (216, 305)	11.1 (11.3, 14.0)	6.06 (5.77, 7.44)
	No (%)	15	187 (154, 245)	6.8 (5.5, 9.3) ***	7.42 (6.02, 9.61) *
**Neonate**	Yes (%)	85	311 (306, 445)	17.4 (17.0, 18.2)	5.57 (5.40, 6.53)
	No (%)	15	213 (134, 391)	13.5 (12.0, 15.6) ***	7.01 (5.34, 10.33) *

Data are geometric mean (95% CI); * *p*-value compared to geometric mean in the respective group(s); * *p* < 0.05; ** *p* < 0.01; *** *p* < 0.001.

### 3.2. Maternal B12 and Metabolic Risk of Offspring

**Figure 1:**
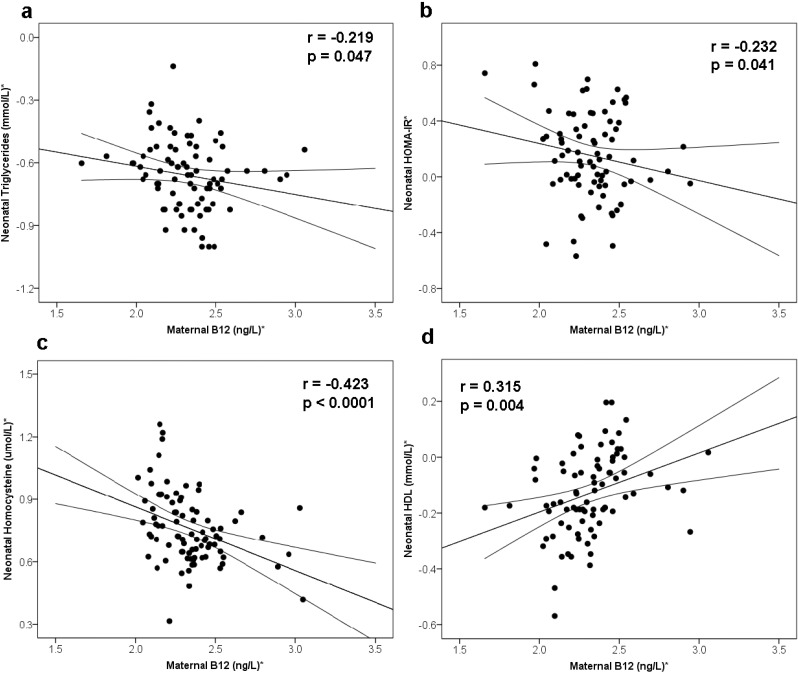
Correlation between maternal B12 (adjusted for age and BMI) and metabolic risk factors of neonates. (**a**) Maternal B12 and neonatal triglycerides, (**b**) Maternal B12 and neonatal Homeostasis Model Assessment 2-Insulin Resistance (HOMA-IR), (**c**) Maternal B12 and neonatal homocysteine, (**d**) Maternal B12 and neonatal HDL. * Log-transformed for statistical comparisons.

Maternal B12 adjusted for age and BMI was inversely associated with metabolic risk factors such as triglycerides (*r* = −0.219; *p* = 0.047), HOMA-IR (*r* = −0.232; *p* = 0.041), homocysteine (*r* = −0.423; *p* = 0.0001) and positively with HDL-cholesterol (*r* = 0.315; *p* = 0.004) (Figure 1a–d) in the offspring. Despite similar birth weight, offspring of low B12 mothers had significantly lower HDL-cholesterol, higher triglycerides and homocysteine than those of normal B12 mothers (Table 2). Multiple regression analysis was carried out to assess whether maternal B12 independently associated with these metabolic risk factors in the offspring by adjusting for likely confounders. The model included maternal age, parity, smoking, folic acid supplement use, BMI, glucose, insulin, folate and homocysteine as independent variables. In addition, for offspring’s lipid parameters, respective maternal variable was also included in the model (maternal triglycerides for offspring’s triglycerides, *etc.*). After all these adjustments, maternal B12 was independently associated with the offspring’s HDL and homocysteine. Though similar trends were seen for the triglycerides and HOMA-IR, these were not statistically significant. No sex-specific changes were seen in any of these analyses (data not shown). Maternal B12 explained 5.1% of the variation in offspring’s HDL and 10.6% in homocysteine (Table 4).

**Table 4:** Association of maternal B12, folate and homocysteine with neonate metabolic risk factors.

Maternal Variable (SDS)	Neonate’s Metabolic Risk Factors (SDS)																				
	Triglycerides *			Cholesterol *			HDL *			LDL *			Insulin *			Glucose *			Homocysteine *		
Maternal B12 *	β	95% CI	*p*	β	95% CI	*p*	β	95% CI	*p*	β	95% CI	*p*	β	95% CI	*p*	β	95% CI	*p*	β	95% CI	*p*
Model 1	−0.148	(−0.38, 0.09)	0.210	0.109	(−0.11, 0.33)	0.317	0.296	(0.07, 0.52)	0.010	−0.044	(−0.26, 0.17)	0.691	0.070	(−0.21, 0.21)	0.516	−0.005	(−0.22, −0.21)	0.960	−0.381	(−0.58, −0.18)	<0.001
Model 2	−0.086	(−0.38, 0.21)	0.562	0.178	(−0.08, 0.44)	0.173	0.294	(0.05, 0.54)	0.018	0.070	(−0.26, 0.39)	0.672	−0.063	(−0.17, 0.29)	0.593	−0.088	(−0.14, −0.31)	0.438	−0.200	(−0.35, −0.05)	0.009
Model 3	−0.079	(−0.39, 0.23)	0.609	0.170	(−0.09, 0.43)	0.198	0.295	(0.08, 0.51)	0.009	0.056	(−0.28, 0.39)	0.736	−0.062	(−0.17, 0.29)	0.602	−0.093	(−0.12, 0.30)	0.378			
Maternal Folate *																					
Model 1	−0.109	(−0.33, 0.11)	0.326	−0.210	(−0.42, 0.001)	0.051	−0.025	(−0.25, 0.19)	0.825	−0.173	(−0.38, 0.04)	0.111	0.040	(−0.17, 0.25)	0.705	−0.124	(−0.33, 0.09)	0.243	−0.327	(−0.53, −0.13)	0.002
Model 2	−0.084	(−0.43, 0.26)	0.625	−0.204	(−0.49, 0.08)	0.160	−0.236	(−0.51, 0.04)	0.091	−0.079	(−0.40, 0.24)	0.625	0.133	(−0.15, 0.42)	0.357	−0.035	(−0.31, 0.24)	0.799	−0.004	(−0.19, 0.18)	0.966
Model 3	−0.093	(−0.45, 0.26)	0.600	−0.200	(−0.49, 0.09)	0.169	−0.209	(−0.46, 0.04)	0.099	−0.082	(−0.41, 0.24)	0.616	0.136	(−0.15, 0.43)	0.353	−0.039	(−0.29, 0.22)	0.763			
Maternal Homocysteine *																					
Model 1	0.218	(−0.00, 0.44)	0.050	0.290	(−0.07, 0.51)	0.009	0.119	(−0.13, 0.36)	0.336	0.265	(0.06, 0.47)	0.013	0.001	(−0.21, 0.21)	0.993	0.121	(−0.09, 0.34)	0.269	0.752	(0.62, 0.89)	<0.001
Model 2	0.137	(−0.19, 0.47)	0.473	0.206	(−0.08, 0.49)	0.156	0.093	(−0.23, 0.41)	0.556	0.224	(−0.09, 0.54)	0.160	0.037	(−0.23, 0.31)	0.785	0.057	(−0.21, 0.32)	0.669	0.696	(0.52, 0.87)	<0.001
Model 3	0.126	(−0.23, 0.48)	0.474	0.230	(−0.07, 0.53)	0.129	0.158	(−0.13, 0.45)	0.278	0.248	(−0.08, 0.57)	0.130	0.043	(−0.24, 0.32)	0.759	0.089	(−0.16, 0.34)	0.469			

* Log transformed for statistical comparisons. β represents SDS change in the dependent variable per SDS change in the independent variable. Model 1: unadjusted; Model 2: Maternal age, BMI, glucose, insulin, parity, folic acid supplement use, smoking, vitamin B12, folate and homocysteine; Model 3: Model 2 + respective maternal variable such as a—Model 2 + maternal triglycerides, b—Model 2 + maternal cholesterol, c—Model 2 + maternal HDL, d—Model 2 + maternal LDL, e—Model 2 + maternal insulin, f—Model 2 + maternal glucose, g—Model 2 + maternal homocysteine.

### 3.3. Maternal Folate and Homocysteine and Metabolic Risk of Offspring

Maternal folate negatively associated with offspring’s cholesterol (*r* = −0.214; *p* = 0.045), LDL (*r* = −0.233; *p* = 0.030) and homocysteine (*r* = −0.346; *p* = 0.001). Maternal homocysteine positively associated with offspring’s triglycerides (*r* = 0.239; *p* = 0.030), cholesterol (*r* = 0.247; *p* = 0.022) and LDL (*r* = 0.244; *p* = 0.026), however, these associations diminished after adjusting for all likely confounders (Table 4).

## 4. Discussion

Our study is the first to show that maternal vitamin B12 levels adversely associated with markers of metabolic risk at birth, in particular lipid profiles. Our observed rates of low B12 status in mothers (40%) is common at the time of delivery though it is not as high as in the South Asian population [10,11,26]. Haemodilution and increased nutrient demand by the growing fetus [27] are known contributors to low B12 levels during pregnancy. In addition, consumption of processed foods, improving hygiene and reheating of cooked food, all known to reduce the bioavailable B12 in food products, could have contributed to lower B12 levels in this population [20,28]. The presence of higher homocysteine in the low B12 group suggest that these low levels are clinically significant and represent true insufficiency at the tissue level.

Our findings show that low maternal B12 status was associated with offspring’s insulin resistance, lower HDL and higher triglycerides (Figure 1a,b,d). However, when multivariate analysis was used to assess the effect of B12 across the spectrum, only HDL was statistically significant after adjusting for the possible confounders (Table 4). In support of this, adverse lipid profile (higher total cholesterol and triglycerides) was noticed in rats born to vitamin B12 restricted dams [18,19]. In addition, we have recently demonstrated that adipocytes cultured in low B12 condition showed increased cholesterol levels and was due to hypomethylation of cholesterol transcription factor (SREBF1 and LDLR) [29]. The clinical findings observed in this study thus add evidence that low maternal B12 status adversely affects lipid profile in the offspring. We did not see any significant association between maternal and neonatal lipids (data not shown). While this was surprising, it was similar to other observations, where only lipids from GDM mothers associated with foetal lipids and not from non-GDM mothers [30,31].

Our study also showed that maternal B12 showed a stronger inverse association with neonatal homocysteine than folate (Supplementary Figure S2a,b). In multiple regression analysis, after adjusting for the possible confounders, only maternal B12 and not folate, was independently associated with neonatal homocysteine (Table 4). The association between maternal folate and neonatal homocysteine became insignificant, when maternal homocysteine was added in the stepwise regression model (Table 4). This suggests that the effect of folate on neonatal homocysteine is likely to be mediated through maternal homocysteine while the effect of B12 could be partly independent of maternal homocysteine. Similar to our findings, Molloy *et al.* showed in an Irish population that low maternal B12 levels predicted hyperhomocysteinemia in both the newborns and the mothers [32]. Thus, our findings confirm that in folate replete populations, B12 is the strongest driver of homocysteine [10], an established metabolic risk factor [33]. Our study also showed that the BMI was higher in the low B12 group (Table 2). Similar observations were seen other studies [29,30,31]. The cause and effect of this relationship is not known. Theoretically this could have contributed to higher maternal lipids and in turn higher lipids in cord blood. However, we did not see any correlation between maternal and cord lipids and our regression analysis adjusted for maternal lipids (Table 4).

The plausible biochemical reasons that low maternal B12 status increase the metabolic risk in the offspring might be, firstly, in the cytoplasm, vitamin B12 acts as a cofactor for conversion of homocysteine to methionine, the direct precursor of *S*-adenosylmethionine (SAM) which is the common donor required for methylation of DNA, protein and lipids [10,26,34]. Secondly, in mitochondria, vitamin B12 also acts a cofactor for the conversion of methylmalonyl Co-A (MM-CoA) to succinyl Co-A. Thus, low vitamin B12 causes higher MM-CoA levels. This in turn can inhibit carnitine palmitoyl transferase-1 (CPT-1), the rate-limiting enzyme for fatty acid β-oxidation, thereby increasing lipogenesis [10,35]. As these mechanisms involve methylation of DNA, this might lead to higher metabolic risk in the offspring by adverse epigenetic programming in addition to directly affecting β-oxidation of fatty acids. *In vivo* and interventional studies are required to identify the exact mechanisms and prove the causality.

Similar to B12, low maternal folate levels also showed adverse correlations with the metabolic risk markers of the offspring but these differences disappeared in regression models. Women with highest B12 and folate levels gave birth to children with lowest homocysteine levels compared to those with lowest B12 and folate levels (7.80 *vs.* 4.85 μmol/L, *p* < 0.001; Supplementary Table S1). Taken together, these findings suggest that optimising the circulating levels of these two B vitamins during pregnancy, is likely to be beneficial to the offspring.

Strengths and limitations: Our study is cross sectional and from a single-centre. However, this is the first study to report the associations between maternal B12 and lipid profiles in the offspring. A prospective cohort of women from before or early pregnancy would have been a better model. As the pathophysiological link, if any, between maternal nutrient status and offspring metabolic risk seem to happen earlier in pregnancy, such longitudinal study would have strengthened our findings [9,10]. Our findings call for such studies to be conducted urgently. Studies have reported that B12 levels progressively decline during pregnancy [36]. Therefore, the effect size we observed during late pregnancy might have been an overestimate if early pregnancy samples were tested. We did not use the microbiological assay for B12 measurements, which is known to be more sensitive at the lower levels of B12. This may have underestimated the rate of low B12 status [37] and in turn, the association with the metabolic risk factors in the offspring. We did not have a detailed socioeconomic status of the participants. It is known that lower socioeconomic status is an important confounder of adverse lipid profiles and BMI but the link between socioeconomic status and B12 is not known. Therefore, this is also a limitation of our observation and future studies should collect detailed socioeconomic status. Finally, although our sample size was adequate to demonstrate the low B12 status, it was probably too small to demonstrate the independent associations between maternal folate and homocysteine status and cord blood lipids.

In summary, our study shows that maternal vitamin B12 plays an important role in lipid metabolism in the offspring and that their restriction *in utero* may predispose them to the increased metabolic risk. However, these findings need to be replicated, ideally in a larger cohort of pregnant women from early pregnancy. In addition, *in vivo* and interventional studies are required to prove the exact mechanisms and the potential causal link. If proven, optimizing B12 levels of young women around the peri-conceptional period, could offer novel opportunities to reduce the burden of obesity and related metabolic disorders of the next generation.

## Supplementary Files

Supplementary File 1
