# Supplementary material for: Low Maternal Vitamin B12 Status Is Associated with Lower Cord Blood HDL Cholesterol in White Caucasians Living in the UK
Source: Nutrients. 2015 Apr 2;7(4):2401–14. doi: 10.3390/nu7042401 (PMC4425151; doi:10.3390/nu7042401)
Supplement: Supplementary File 1 [file nutrients-07-02401-s001.docx]

Supplementary Information

**Table S1.** Vitamin B12, folate and homocysteine in mothers and neonates according to quartiles of maternal B12 and quartiles of maternal folate.

|  | | **Quartiles of Maternal B12 (ng/L)** | | | | ***p* for trend** |
| --- | --- | --- | --- | --- | --- | --- |
|  |  | **Quartile-1  ≤148.5** | **Quartile-2  148.6–211.0** | **Quartile-3 211.1–271.6** | **Quartile-4  ≥271.7** |  |
|  |  |  |  |  |  |  |
| Maternal | Folate (μg/L) | 9.0 (8.0, 12.8) | 9.4 (8.3, 13.2) | 10.5 (9.3, 14.0) | 13.7 (12.7, 17.7) | 0.004 |
|  | Homocysteine (μmol/L) | 8.13 (6.73, 11.9) | 6.01 (5.46, 6.98) | 5.18 (4.68, 6.13) | 5.94 (5.38, 6.88) | 0.002 |
| Neonatal | Vitamin B12 (ng/L) | 190 (169, 233) | 221 (199, 261) | 294 (250, 397) | 561 (473, 844) | <0.001 |
|  | Folate (μg/L) | 15.3 (14.3, 17.0) | 16.7 (15.6, 18.4) | 16.7 (15.7, 18.2) | 18.6 (17.8, 19.9) | 0.001 |
|  | Homocysteine (μmol/L) | 7.8 (6.83, 10.2) | 5.72 (5.18, 6.97) | 4.96 (4.38, 5.98) | 4.85 (4.49, 5.46) | <0.001 |
|  | | **Quartiles of Maternal Folate (μg/L)** | | | | ***p* for trend** |
|  |  | **Quartile-1  ≤6.36** | **Quartile-2  6.37–10.82** | **Quartile-3 10.83–17.50** | **Quartile-4  ≥17.60** |  |
|  |  |  |  |  |  |  |
| Maternal | Vitamin B12 (ng/L) | 187 (163, 237) | 204 (147, 326) | 223 (173, 351) | 260 (217, 383) | 0.061 |
|  | Homocysteine (μmol/L) | 7.73 (6.31, 11.3) | 6.39 (5.59, 7.95) | 6.19 (5.54, 7.35) | 4.91 (4.50, 5.60) | <0.001 |
| Neonatal | Vitamin B12 (ng/L) | 237 (191, 346) | 252 (170, 499) | 323 (257, 564) | 360 (293, 517) | 0.081 |
|  | Folate (μg/L) | 14.6 (13.8, 15.7) | 15.5 (14.5, 16.9) | 17.8 (16.8, 19.2) | 19.6 (19.0, 20.4) | <0.001 |
|  | Homocysteine (μmol/L) | 6.75 (5.88, 8.95) | 5.95 (5.20, 7.66) | 5.84 (4.95, 7.74) | 4.73 (4.42, 5.22) | 0.003 |

Data are geometric mean (95% CI).


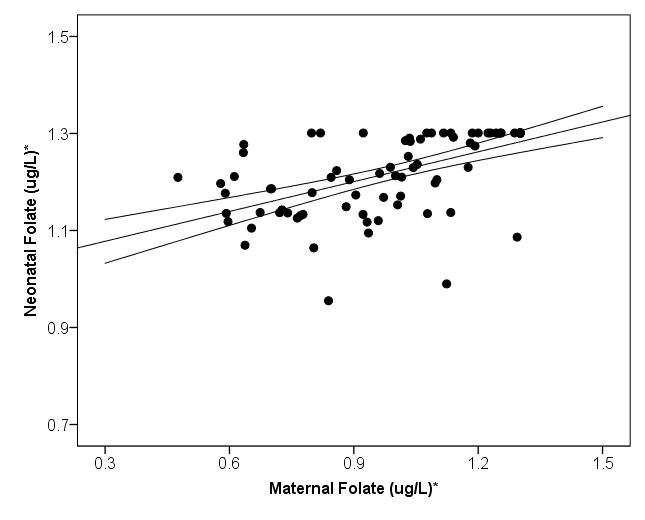

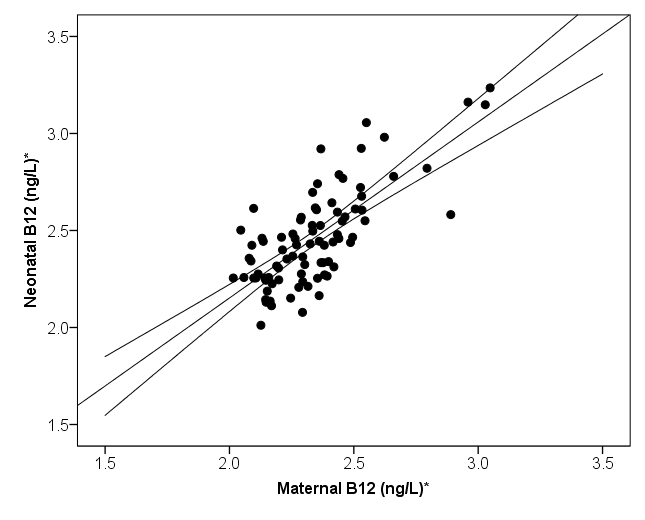


***r* = 0.648**

***p* < 0.0001**

***r* = 0.706**

***p* < 0.0001**

**b**

a

**Figure S1.** *Cont.*

**
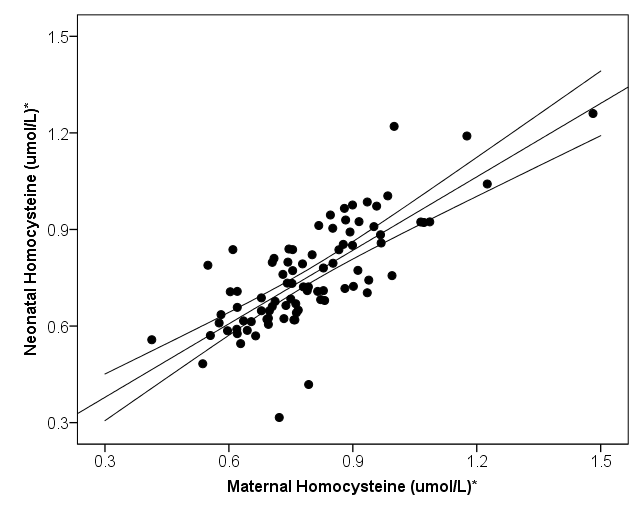
**

***r* = 0.756**

***p* < 0.0001**

**c**

**Figure S1.** Correlation between maternal and neonatal indices (**a**) Maternal B12 and neonatal B12 (**b**) Maternal Folate and neonatal Folate (**c**) Maternal Homocysteine and neonatal Homocysteine. *Log-transformed for statistical comparisons.

**Figure S2.** Correlation between (**a**) Maternal B12 and neonatal Homocysteine (**b**) Maternal Folate and neonatal Homocysteine. *Log-transformed for statistical comparisons.
